# Analysis of color and texture characteristics of cereals on digital images

**DOI:** 10.18699/VJ20.626

**Published:** 2020-07

**Authors:** E.G. Komyshev, M.A. Genaev, D.A. Afonnikov

**Affiliations:** Institute of Cytology and Genetics of Siberian Branch of the Russian Academy of Sciences, Novosibirsk, Russia Kurchatov Genomic Center of the Institute of Cytology and Genetics of Siberian Branch of the Russian Academy of Sciences, Novosibirsk, Russia; Institute of Cytology and Genetics of Siberian Branch of the Russian Academy of Sciences, Novosibirsk, Russia Kurchatov Genomic Center of the Institute of Cytology and Genetics of Siberian Branch of the Russian Academy of Sciences, Novosibirsk, Russia Novosibirsk State University, Novosibirsk, Russia; Institute of Cytology and Genetics of Siberian Branch of the Russian Academy of Sciences, Novosibirsk, Russia Kurchatov Genomic Center of the Institute of Cytology and Genetics of Siberian Branch of the Russian Academy of Sciences, Novosibirsk, Russia Novosibirsk State University, Novosibirsk, Russia

**Keywords:** color, texture, digital images, image analysis, cereal grains, цвет, текстура, цифровые изображения, анализ изображений, зерна злаков

## Abstract

The color of the grain shell of cereals is an important feature that characterizes the pigments and metabolites contained in it. The grain shell is the main barrier between the grain and the environment, so its characteristics are associated with a number of important biological functions: moisture absorption, grain viability, resistance to pre-harvest germination. The presence of pigments in the shell affects various technological properties of the grain. Color characteristics, as well as the appearance of the grain shell are an important indicator of plant diseases. In addition, the color of the grains serves as a classifying feature of plants. Genetic control of the color formation of both grains and other plant organs is exerted by genes encoding enzymes involved in the biosynthesis of pigments, as well as regulatory genes. For a number of pigments, these genes are well understood, but for some pigments, such as melanin, which causes the black color of grains in barley, the molecular mechanisms of biosynthesis are still poorly understood. When studying the mechanisms of genetic control of grain color, breeders and geneticists are constantly faced with the need to assess the color characteristics of their shell. The technical means of addressing this problem include spectrophotometers, spectrometers, hyperspectral cameras. However, these cameras are expensive, especially with high resolution, both spatial and spectral. An alternative is to use digital cameras that allow you to get high-quality images with high spatial and color resolution. In this regard, recently, in the field of plant phenotyping, methods for evaluating the color and texture characteristics of cereals based on the analysis of two-dimensional images obtained by digital cameras have been intensively developed. This mini-review is devoted to the main tasks related to the analysis of color and texture characteristics of cereals, and to methods of their description based on digital images.

## Abstract (ru)

Цвет оболочки зерен злаков – важный признак, характеризующий содержащиеся в ней пигменты и метаболиты. Оболочка зерна служит основным барьером между зерном и внешней средой, поэтому с ее характеристиками связан ряд важных биологических функций: поглощение влаги, жизнеспособность зерна, устойчивость к предуборочному прорастанию. Наличие пигментов в оболочке влияет на различные технологические свойства зерна. Цветовые характеристики, как и внешний вид оболочки зерна, – важный индикатор заболеваний растений. Цвет зерна давно используется в систематике пшеницы для описания ее ботанических разновидностей, и для некоторых систем это одна из основных характеристик. Генетический контроль формирования окраски зерен и других органов растений осуществляется генами, кодирующими ферменты, вовлеченные в биосинтез пигментов, и регуляторными генами. Для ряда пигментов эти гены исследованы достаточно хорошо, однако для некоторых пигментов, например меланина, обусловливающего черную окраску зерен у ячменя, молекулярные механизмы биосинтеза еще слабо изучены. При исследовании механизмов генетического контроля окраски зерен селекционеры и генетики постоянно сталкиваются с необходимостью оценки цветовых характеристик их оболочки. К техническим средствам решения этой задачи относятся спектрофотометры, спектрометры, гиперспектральные камеры. Однако эти камеры дорогостоящие, в особенности с высоким разрешением, как пространственным, так и спектральным. Альтернативой является использование цифровых фотокамер, позволяющих получать высококачественные изображения с высоким пространственным и цветовым разрешением. В связи с этим в последнее время в области фенотипирования растений интенсивно развиваются методы оценки цветовых и текстурных характеристик зерен злаков, основанные на анализе двумерных изображений, полученных цифровыми камерами. Данный мини-обзор посвящен основным задачам, связанным с анализом цветовых и текстурных характеристик зерен злаков, методам их описания на основе цифровых изображений.

## kwd>

The coloration of cereal grain shell is an important trait that characterizes the pigments and metabolites contained there. Violet and blue grain color is determined by anthocyans; yellow color, by carotenoids; and red brown or dark brown, by flavonoids, such as proanthocyanidins and phlobaphenes (Adzhieva et al., 2015; Lachman et al., 2017). The correlation between the shell color and content of the corresponding substances is experimentally demonstrated. Significant correlations of the kernel shell color and the content of phenols, flavonoids, and antioxidant capacity have been observed (Shen et al., 2009). The contents of phenols, flavonoids, anthocyans, β-carotenoids, and luteins significantly differ between the maize grains with different colors (Žilić et al., 2012). Flavonoids, anthocyans, and carotenoids possess several valuable properties. They are antioxidants, and influence the nutritional value. For example, addition of the wheat seed coats with a purple pericarp or blue aleurone layer to flour improves the quality of bakery products owing to flavor, texture, and color characteristics (Machálková et al., 2017). Correspondingly, the varieties and lines with different grain coloration recently cause a strong interest of the food industry (Khlestkina et al., 2017; Corrêa et al., 2019).

The kernel shell is the main barrier between the grain and environment; correspondingly, a set of important biological functions are associated with the shell properties, including, water absorption, grain viability, and resistance to pre-harvest sprouting (Souza, Marcos-Filho, 2001). The pigments in the grain shell influence manifold grain technological properties. In particular, phlobaphenes (condensed tannins), coloring the pericarp red, have a positive effect on the duration of grain dormancy, thereby preventing its pre-harvest sprouting (Flintham et al., 2002). That is why the wheat genotypes with red-colored kernels are used in breeding as a donor of the genes controlling the resistance to pre-harvest grain germination (Krupnov et al., 2012; Fakthongphan et al., 2016). The shell color of rice kernels (the intensities of red, green, and blue color components) correlates with the grain quality characteristics, such as kernel transparency and the share of broken kernels, in a statistically significant manner (Septiningsih et al., 2003).

The color characteristics and the external appearance of the kernel shell are important indicators of plant diseases. For example, fusariosis manifests as a pinky or bluish coloration on the wheat and barley kernel shell (McMullen et al., 1997). Characteristic of another disease, kernel black-point, is dark discoloration of the embryo side of grains (Draz et al., 2016).

Grain coloration may also serve as a trait in plant classification. As early as the late 19th century, F. Körnicke suggested using the grain color for description of wheat botanical varieties (Körnicke, Werner, 1885). The N.I. Vavilov All-Russian Institute of Plant Genetic Resources classifies the wheat botanical varieties using the system in which the grain color is one of the major traits (Dorofeev et al., 1979).

The coloration of both the kernels and other plant organs is controlled by the genes coding for the enzymes involved in biosynthesis of pigments, as well as by regulatory genes (Khlestkina, 2014; Lachman et al., 2017; Shoeva et al., 2018). The corresponding genes are well studied for several pigments, including even their complete nucleotide sequences and their positions in the genome. However, the molecular mechanisms of biosynthesis are still rather vague for some pigments, in particular, melanin, which determines black color of barley kernels (Glagoleva et al., 2017; Shoeva, 2018). 

When studying the mechanisms underlying genetic control of grain color, breeders and geneticists constantly face the necessity of estimating the color characteristics of their shells. Several technical tools allow this problem to be solved, first and foremost, spectrophotometers, able to characterize both the chromatic and textural characteristics of kernels with a high accuracy. Spectrophotometers have been long and successfully used and serve as a standard for estimating the color of biological objects (Black, Panozzo, 2004; Garg et al., 2016; Machálková et al., 2017). Another approach is provided by spectrometers with the wavelength range covering both visible and nearinfrared regions (hyperspectral cameras of visible and near-infrared ranges) (Black, Panozzo, 2004; ElMasry et al., 2019). However, these cameras are very expensive, especially, with a high spatial and spectral resolution. An alternative is digital cameras capturing high-quality images with a high spatial and color resolution. The price of digital cameras are constantly decreasing and they are now widely available, while even an amateur camera allows for capturing high-resolution and high quality images. In this regard, the methods for evaluating the color and texture characteristics of cereal grains based on analysis of two-dimensional digital images have been intensively developed recently in the field of plant phenotyping.

This brief review focuses on the main problems related to the analysis of color and texture characteristics of cereals and methods of their description utilizing digital images.

## The tasks associated with the analysis of color and texture characteristics in kernel images

One of the relevant problems in the analysis of digital images of grains is related to classification. The particular tasks of classification may be different. For example, it is necessary to classify grains according to their color and surface texture into several different genotypes (Pourreza et al., 2012; Olgun el al., 2016). Frequently, the characteristics of size and shape are added to the color and texture parameters (Majumdar, Jayas, 2000; Chaugule, Mali, 2014; Sabanci et al., 2016).

Another tightly associated problem is to assort the kernels according to color and surface texture (Pearson, 2010); in particular, sorters are designed for mass screening of a large number of grains to separate the sound grains from waste and damaged grains. M. Huang et al. (2015) reviewed the current developments on the seed quality and safety tests based on image analysis, including hyperspectral ones, and Z. Gong et al. (2015) briefly describe the approaches, engineering included, to the seed quality inspection.

Sometimes the grains are classified only by their color (red or white). In particular, M.S. Ram et al. (2002) used spectrophotometer and spectrometer to design a procedure for determining the color of kernel shell in red and white wheats. T.N. McCaig et al. (1993) classified the wheat into red-grained and white-grained cultivars using the spectrophotometry data for 262 genotypes of both soft and hard wheats. Analysis of the color characteristics also makes it possible to identify the kernels affected by pathogens (Ahmad, 1999; Goriewa-Duba et al., 2018) or mechanically damaged (Delwiche et al., 2013). Note that machine learning and artificial intelligence techniques are also frequently used along with the image analysis in solving the relevant problems (Patrício, Rieder, 2018); however, the description of these methods is beyond the area of our review.

## Color coding systems

The color of the surface is a characteristic of its spectral reflectivity, which is determined by many factors, such as absorption of radiation of a light source at different wavelengths, its reflection, and scattering (Forsyth, Pons, 2004). Spectrometers give the fullest estimate of the reflection and absorption characteristics in different ranges of wavelengths. As for the most of the digital cameras, their sensors respond to reflected radiation in the visible wavelength range (400–780 nm). Note that the color perception by the human eye has its specific features associated with its structure: there is no one-to-one correspondence between the surface color perception by the eye and the spectral characteristics of this surface; for example, the same shade of gray can be reproduced by the reflected radiation with completely different intensities for different wavelengths.

When studying the human perception, it was found out that three main colors – red, green, and blue – are sufficient to get the overall set of colors perceived by humans by mixing them in different proportions (Forsyth, Pons, 2004). This inference is confirmed by the structure of the human eye itself since the eye retina comprises three types of receptors (retinal cones) responsible for color vision.

Different models (color spaces) have been elaborated to digitally represent colors. Color model specifies the system of coordinates that unambiguously determines colors. Several different color models have been developed to provide the best method of color description for TV, photo, video, and color printing. The following systems are most frequently used when analyzing digital images of plants.

The RGB color model is the most well known color space, encoding a broad array of colors by relative intensities of its three components: red (R), green (G), and blue (B). These components are described by integers, most frequently from 0 to 256. The higher the values, the higher is the intensity of color (luminance). The colors with equal values of the components are the shades of gray. This representation is used mainly in computer screens and digital cameras.

The HSV (HSB) model is a color space also using three color components, proposed in the mid-1970s. The hue component (H) varies from 0 to 360; the values close to 0 and 360 correspond to red; close to 60, to yellow; 120, to green; 180, to cyan; 240, to blue; and 300, to magenta. Saturation (S) is the larger, the more saturated is the color tone, while small values of this parameter correspond to the shades of gray. Brightness (value, B/V) takes on the smaller values for the dark colors and larger, for bright ones. One of the shortcomings of the HSV and RGB consists in that the number of saturation and color tint levels perceptible to eye in these spaces decreases when brightness approaches zero

The CIE L*a*b* space, proposed in 1976 by the International Commission on Illumination (CIE), was designed to approximate the human vision and to provide perceptual uniformity. Similar to HSV, the brightness component in CIE L*a*b* (L* component) is separated from the chromatic component of color (Pathare et al., 2013) and is an approximate estimate of brightness. The a* parameter takes on the positive values for reddish tints and negative values for greenish ones; the b* parameter is positive for the yellowish tints and negative for the bluish ones. This color model is widely used in software solutions for image processing and color correction. The CIE L*a*b* space is used for assessing the color characteristics in spectrophotometers.

The characteristics of other color spaces with their description are available in specialized literature on image analysis (Fisenko V.T., Fisenko T.Yu., 2008; Domasev, Gnatyuk, 2009). The components of the same color in different systems are linked by transformation rules, so that knowing the values of the chromatic components for a color tint in one space, the corresponding values for another space are obtainable. For example, the values for the RGB components make it possible to compute the values for the HSV components and vice versa. This allows the color representation for a particular image to be selected depending on the particular task

When solving a problem of machine vision and analysis of chromatic characteristics, the HSV and L*a*b* color models are of the principal interest since these systems represent colors in the same terms as a human does when describing a color, namely, hue, saturation, and brightness (lightness).

## Analysis of color characteristics of kernels

The images used for analyzing kernels are as a rule captured by digital cameras in the RGB space shot under laboratory conditions using a controlled illumination. The kernels in images are typically placed onto a contrast background at a distance from one another (Sabanci et al., 2017; Goriewa-Duba et al., 2018). This protocol makes it possible to analyze not only the color and surface texture characteristics, but also the kernel shape and size. Moreover, bulk specimens are used in some studies (Pourreza et al., 2012; Olgun et al., 2016) in which the kernels lie in a dense grain touching one another. As a rule, the textural and chromatic characteristics of the bulk specimen are assessed in this approach rather than individual kernels.

In the case the individual kernels are analyzed, first, their local images are isolated in the integral image. For this purpose, the images are preprocessed (using despeckle and removal of noise and foreign objects) and segmented to identify the regions of the image that correspond to individual kernels. Then, the quantitative traits available from images are extracted from these regions. Note that it is rather difficult to control the illumination conditions, especially when the images are captured outside laboratory (Berry et al., 2018). Correspondingly, color correction procedure using color patterns (a set of cards with cells of specified standard colors) is helpful (Berry et al., 2018; Genaev et al., 2019; Alemu et al., 2020).

In an image, the regions corresponding to individual kernels comprise hundred of pixels, each displaying its own color characteristics in a selected color space (for example, three values of the R, G, and B components). That is why statistical characteristics of color components are most frequently used for description of the color of these objects. First and foremost, the histograms of pixel distribution according to the intensity of each color component independently of the other components and the location of pixels in the image are computed. The histograms are used to calculate the other parameters, such as the mean value, variance, asymmetry, and the kurtosis of pixel intensities for each color component (Ahmad et al., 1999; Majumdar, Jayas, 2000). These values are further utilized to describe the color properties of kernels.

In particular, T. Pearson and D. Brabec (2008) developed a system of machine vision for an automated estimation and sorting of the kernels of wheat and other cereals in a real-time mode. The images with a resolution of 640×480 pixels were captured with a digital camera and transferred to a PC, which, after classification, output a signal to an air valve to correspondingly sort the kernels. The intensity histograms as well as the mean and standard deviations of the RGB channel intensities (in total, 198 characteristics for each kernel) were used for classification by linear discriminant analysis. The accuracy of the system when classifying red and white kernels of hard wheat was 94 to 99 % depending on the wheat cultivar, feeding rate, and number of classification characteristics.

N.S. Visen et al. (2002) compared the accuracy of different architectures of simple and specialist neural networks in the classification of cereals. Morphological and chromatic characteristics of wheat, barley, oat, and rye kernels calculated using color images captured with a CCD camera were used as the input data. The gray features: mean, median, mode, and standard deviation of gray-level values of the objects in the image – were extracted and used as the input data. The best mean classification accuracy of 98 % was obtained using specialist probabilistic neural networks.

K. Goriewa-Duba et al. (2018) used the digital images of the kernels of six wheat species acquired with a flatbed CCD scanner to analyze the shape and color. They assessed the effect of grain colonization by endophytic fungi on the color of the seed coat as well as estimated the wheat subspecies with a high genetic variation. The images were analyzed using the ImageJ software to assess the shape characteristics, such as area, perimeter, Feret diameter, circularity, aspect ratio, roundness, and solidity. The color descriptors included the mean values for the RGB, HSI, and L*a*b* channels. The principal component analysis of the kernels with different genotypes has shown that their color characteristics significantly contribute to the first variance component and are among the most important when classifying wheats into different genotypes.

A. Alemu et al. (2020) analyzed the genome-wide associations of nucleotide substitutions (GWAS) in the population of 192 hard wheat (Triticum durum) genotypes from Ethiopia with grain shape and color traits. Grain length and width were used to describe the kernel shape and the mean values of the L*a*b* components to describe the color. In total, 11 quantitative trait loci (QTLs) were detected for the color characteristics; the locus for the a* component resides on chromosome 2A; five loci for the b* component, on chromosomes 1B, 3A, 4B, 5A, and 7B; and five for the L* component, on chromosomes 1A, 2A, 7A, and 7B.

## Characteristics of the image texture used when analyzing kernels

Another characteristic of the surface is its texture, which is the image component that reflects the visual properties of these surfaces or objects (bumpiness and the presence of regular patterns). The concept of texture is difficult to formalize since it to a considerable degree depends on the scale and has not any limitations on the basis of which it is formed. A leaf in an image is an object and the foliage is a texture. It is possible to separate simple textures that are formed of ordered patterns or textons (Forsyth, Pons, 2004). A distinctive feature of a simple texture is its regularity and repeated or partially reproduced elements on a certain surface or object. Other textures may have a considerably more complex structure.

The approaches more intricate as compared with the color analysis are used to describe textures since the texture is characterized by mutual spatial arrangement of pixels with different intensities of their color components. Both the color and texture characteristics can be determined utilizing statistical methods to assess the parameters of the histograms of the initial image, such as, the mean, variance, asymmetry, and kurtosis. For simplicity, the images are described in the gray scale, i. e., the description is reduced from pixel color characteristics (three components) to its total intensity alone (one component, I(x, y), where x and y are the coordinates of pixel). The gray level co-occurrence matrix (GLCM) is used for this purpose (Supplementary Materials, Table 1, Eq. (1))^1^, which is a second order histogram (Haralick et al., 1973). The second order means that the matrix describes the distribution of intensities for the pairs of pixels of an image with specific values. Thus, the combinations of intensities for these pairs are taken into account. See V.G. Astafurov et al. (2014) for an example of computation of a GLCM. The GLCM is then used to extract the statistics for the distribution of its elements, such as uniformity, homogeneity, moments of inertia, correlation, different mean values, variance, and entropy (see Supplementary Materials, Table 2) (Majumdar, Jayas, 1999).

Supplementary Materials are available in the online version of the paper: https://vavilov.elpub.ru/jour/manager/files/SupplKomyshev_engl.pdf


The gray level run length matrix (GLRM; see Supplementary Materials, Table 1, Eq. (2)) is constructed based on the information about the run length of the pixels with equal intensity (Galloway, 1975). These run lengths can be specified by different levels of intensities and the traversal direction from one pixel to another. GLRM allows for computation of the statistics, such as inhomogeneity of gray level, inhomogeneity of run lengths, coefficient of runs, entropy, inverse moment of short runs, moment of long runs, and other characteristics (Haralick et al., 1979).

The third approach relies on the model-based interpretation of texture, for example, a method based on autoregressive model parameters in which the intensity of a pixel is predicted as the weighted sum of four intensities of the neighboring pixels (Szczypiński et al., 2015). Several methods for texture description utilize Fourier, Gabor, or wavelet transform to characterize the spatial arrangement of the pixels of different intensities in the image from its frequency characteristic or wavelet components (Szczypiński et al., 2009). In general, the above briefed characteristics make it possible to form over a hundred of digital traits of image textures. As a rule, only part of these characteristics is used in the relevant literature.

An example of the use of texture characteristics in kernel analysis is the study by A. Pourreza et al. (2012). Images of nine wheat cultivars in a container illuminated with a fluorescent lamp were analyzed. The matrices (GLCM and GLRM) were computed for the gray scale images. Three additional characteristics were used; these characteristics are determined by the difference between the intensity of the central pixel from the intensities of the neighboring pixels in a 3×3 matrix. The local similarity patterns (LSPs; see Supplementary Materials, Table 1, Eq. (3)) are calculated from the difference in the intensities between the central and neighboring pixels. If the difference is below the SRR threshold, the LSP of the neighboring pixel is set equal to unity; otherwise, zero. A clockwise traversal of eight pixels gives a vector of zeros and unities, which characterizes the correspondence of the intensity of the central pixel and all its neighbors. Another parameter in this work is local binary patterns (LBPs; see Supplementary Materials, Table 1, Eq. (4)), which were computed taking into account the weight coefficients of the neighboring pixels multiplied by LSP vector components. Finally, one more texture characteristics was the local similarity number (LSN; see Supplementary Materials, Table 1, Eq. (5)), which is the number of pixels with an intensity similar to that of the central pixel in the square of N×N.

Then, different statistics were calculated for the above textural features (mean, standard deviation, entropy, etc.; in total, 131 features). Some of them were based on the histogram gray level quantification (25 histogram bands). As was demonstrated, the textural features were most effective in classifying the cultivars as compared with the other characteristics. Six of the nine cultivars were identified with a 100 % accuracy; two of the remained cultivars were identified with 96 % accuracy. The use of the characteristics obtained from the LBP, LSP, and LSN matrices improved the classification accuracy as compared with the earlier studies. In total, 54 % of the 50 main textural features were selected from LBP, LSP, and LSN groups. The authors also conclude that the characteristics of feature distribution considerably contributed to identification of wheat cultivars.

K. Sabanci et al. (2017) describes a machine vision system for distinguishing of kernels between the durum and bread wheats. The used visual characteristics include size (length, width, perimeter, and area), color (R, G, and B), and texture (contrast, correlation, energy, homogeneity, and entropy); in addition, nine characteristics were calculated from the main ones. In tests, the simplified classifier identifies the grain type with an accuracy of 99.46 % and sorts the wheat kernels with an accuracy of 100 %. For training and verification, images of 200 wheat kernels (100 of bread wheat and 100 of durum wheat) were captured by a high-resolution camera.

## Conclusion

Spectrophotometers, spectrometers, and hyperspectral cameras are efficient and reliable tools for analysis and estimation of cereal kernels. However, they are expensive, especially those with a high resolution, both spatial and spectral. An alternative is digital cameras capturing highquality images with a high spatial and color resolution. Although the precieved spectrum of the currently available digital cameras is limited, the studies have shown that they can be effectively used as a reliable and precise tool for solving manifold applied problems. A high spatial and color resolution of such cameras makes it possible to analyze the textural characteristics of cereal kernels in detail. The textural characteristics are supplemented with color characteristics represented in different color models.

Thus, the use of color and textural characteristics in the analysis of digital images of cereal kernels allow for an efficient resolution of several important problems in their classification, sorting, and identification of diseases.

## Conflict of interest

The authors declare no conflict of interest.
